# Nothing Regular about the Regulins: Distinct Functional Properties of SERCA Transmembrane Peptide Regulatory Subunits

**DOI:** 10.3390/ijms22168891

**Published:** 2021-08-18

**Authors:** Nishadh Rathod, Jessi J. Bak, Joseph O. Primeau, M’Lynn E. Fisher, Lennane Michel Espinoza-Fonseca, Mary Joanne Lemieux, Howard S. Young

**Affiliations:** 1 Department of Biochemistry, University of Alberta, Edmonton, AL T6G 2H7, Canada; nishadh@ualberta.ca (N.R.); Jessi.Bak@dal.ca (J.J.B.); jprimeau@ualberta.ca (J.O.P.); mlynn1@ualberta.ca (M.E.F.); mlemieux@ualberta.ca (M.J.L.); 2 Center for Arrhythmia Research, Department of Internal Medicine, Division of Cardiovascular Medicine, University of Michigan, Ann Arbor, MI 48109, USA; lmef@med.umich.edu

**Keywords:** calcium transport, sarco-endoplasmic reticulum, SERCA, phospholamban, sarcolipin, DWORF, myoregulin, another-regulin, endoregulin

## Abstract

The sarco-endoplasmic reticulum calcium ATPase (SERCA) is responsible for maintaining calcium homeostasis in all eukaryotic cells by actively transporting calcium from the cytosol into the sarco-endoplasmic reticulum (SR/ER) lumen. Calcium is an important signaling ion, and the activity of SERCA is critical for a variety of cellular processes such as muscle contraction, neuronal activity, and energy metabolism. SERCA is regulated by several small transmembrane peptide subunits that are collectively known as the “regulins”. Phospholamban (PLN) and sarcolipin (SLN) are the original and most extensively studied members of the regulin family. PLN and SLN inhibit the calcium transport properties of SERCA and they are required for the proper functioning of cardiac and skeletal muscles, respectively. Myoregulin (MLN), dwarf open reading frame (DWORF), endoregulin (ELN), and another-regulin (ALN) are newly discovered tissue-specific regulators of SERCA. Herein, we compare the functional properties of the regulin family of SERCA transmembrane peptide subunits and consider their regulatory mechanisms in the context of the physiological and pathophysiological roles of these peptides. We present new functional data for human MLN, ELN, and ALN, demonstrating that they are inhibitors of SERCA with distinct functional consequences. Molecular modeling and molecular dynamics simulations of SERCA in complex with the transmembrane domains of MLN and ALN provide insights into how differential binding to the so-called inhibitory groove of SERCA—formed by transmembrane helices M2, M6, and M9—can result in distinct functional outcomes.

## 1. Background on SERCA Regulation

The sarco-endoplasmic reticulum calcium pump (also known as Ca^2+^-ATPase or SERCA) is ubiquitously expressed in the SR/ER membranes of all eukaryotic cells and tissues. SERCA maintains the low resting-state calcium concentration in the cytosol by transporting calcium against a much higher calcium concentration into the SR/ER lumen. The cytosolic calcium concentration serves as a trigger for the activation of a variety of cellular signaling pathways [1], and SERCA-mediated calcium transport decreases the cytosolic calcium concentration as a trigger for terminating these signaling events [2]. In vertebrates, there are three SERCA genes—SERCA1 (*ATP2A1* gene), SERCA2 (*ATP2A2*), and SERCA3 (*ATP2A3*)—and a variety of splice variants, which differ in their tissue-specific and developmental expression profiles [3]. For the most part, the SERCA isoforms have subtle differences in their structural and functional properties—for example, calcium transport properties including relative calcium affinities and maximal activities—which cater to the specific calcium homeostasis requirements of each cell and tissue type [4]. The exception is the ubiquitous SERCA2b isoform, which is a unique structural and functional splice variant containing an 11th transmembrane helix and a C-terminal extension that resides in the ER lumen [5,6]. This unique modification of SERCA2b imparts the highest calcium affinity of the SERCA isoforms [7] and provides a potential point for regulation from within the ER lumen. Thus, of all the SERCA isoforms, SERCA2b is the only isoform that contains a regulatory domain encoded in the primary structure of the protein.

**Figure 1:**
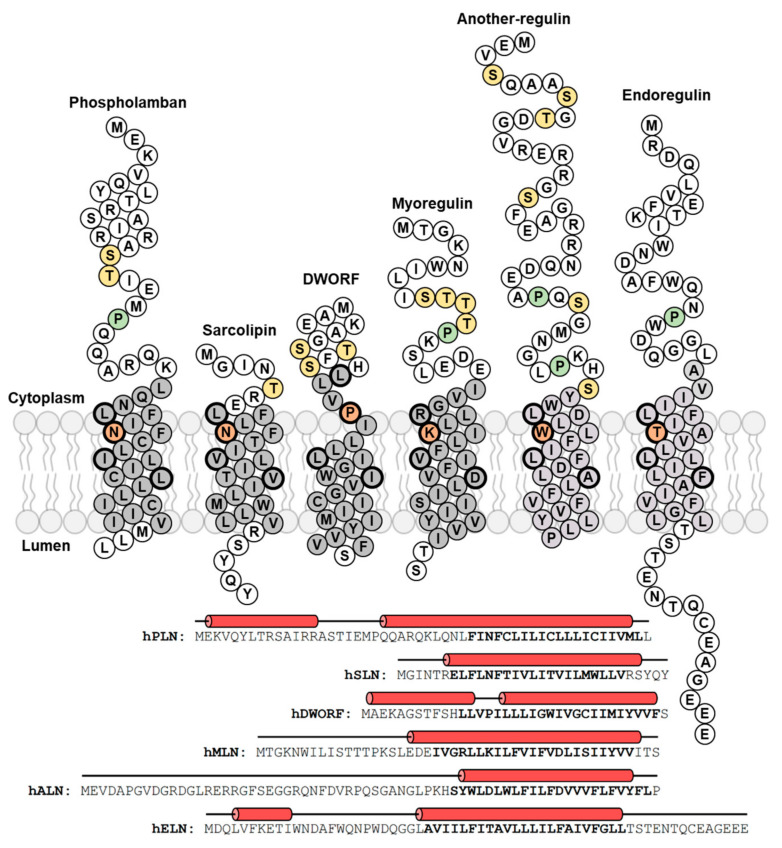
Topology models and sequence alignments for the regulin family of small transmembrane peptides. For the topology models, the transmembrane domains are highlighted in gray, Asn^34^ in PLN and Asn^11^ in SLN are highlighted in orange. Polar residues in MLN, ALN, and ALN that align with Asn^34^ of PLN and Asn^11^ of SLN are also highlighted in orange. Potential phosphorylation sites (yellow) and proline residues (green) are indicated. Pro^15^ of DWORF aligns with Asn^34^ of PLN and Asn^11^ of SLN and is colored as an essential residue (orange). For the sequence alignments, the predicted transmembrane domains are in bold and predicted helical regions are indicated as red cylinders.

SERCA-dependent calcium homeostasis enables a variety of physiological processes, with the contraction (systole) and relaxation (diastole) of cardiac and skeletal muscles being well-known examples. During systole, the cytosolic calcium concentration increases due to the opening of the Ryanodine receptor (RyR) calcium channels and the release of SR calcium stores. During diastole, the cytosolic calcium concentration decreases due to the activity of the SERCA calcium pump. In contrast to the regulatory domain encoded in the SERCA2b isoform, SERCA2a in cardiac muscles and SERCA1a in skeletal muscles are regulated by small transmembrane regulatory subunits called phospholamban (PLN) and sarcolipin (SLN), respectively (Figure 1). PLN is expressed in cardiac, slow-twitch skeletal, and smooth muscles [8] and SLN is expressed in fast-twitch skeletal and atrial muscles [9]. PLN and SLN play critical roles in muscle contractility by physically interacting with SERCA and altering its affinity for calcium [10]. In cardiac muscles, PLN is a calcium-dependent inhibitor of SERCA, which modulates cardiac contractility in response to physiological signals [8]. By comparison, SLN is a calcium-dependent inhibitor of SERCA in skeletal muscles, which has been reported to play a role in contractility and muscle-based thermogenesis [11]. Maximal SERCA inhibition by PLN and SLN occurs within the physiological range of cytosolic calcium (0.1 to ~3.0 µM) and inhibition is relieved by phosphorylation. PLN is the target of β-adrenergic signaling and phosphorylation by protein kinase A [12], calcium/calmodulin-dependent protein kinase II (CaMKII [13]), and protein kinase B (Akt [14]). SLN appears to be a target for phosphorylation by CaMKII [15] and serine/threonine kinase 16 (STK16 [16]). These regulatory mechanisms modulate SR calcium homeostasis and contractility in response to need (e.g., activity, stress, or disease).

Improvements in bioinformatics, proteomics, transcriptomics, and functional analyses have led to the identification of small open reading frames that encode peptides of less than 100 amino acids in length [17,18]. Many of these small open reading frames encode small membrane proteins with a diverse array of physiological functions, tissue distributions, and cellular localizations [19]. While the identification of small membrane proteins continues to expand, relatively few have been thoroughly characterized for their functional and physiological roles. Perhaps the best example of convergence between the ongoing discovery of small membrane proteins and human physiology is the regulation of SERCA-dependent calcium homeostasis. SERCA was long known to be regulated by PLN in cardiac muscles [20] and SLN in skeletal muscles [9]. However, a recent breakthrough revealed that small membrane protein regulators of SERCA are conserved from insects to humans over 550 million years of evolution and they are encoded by small open reading frames within RNAs previously annotated as noncoding [21]. The small transmembrane peptides myoregulin (MLN) and dwarf open reading frame (DWORF) were soon found to be encoded by muscle-specific transcripts in skeletal and cardiac muscles, respectively [22,23]. At present, SERCA is known to be regulated by a family of small membrane protein subunits called the “regulins”. The regulins consist of seven different regulatory subunits including PLN, SLN, sarcolambans (SLB) in insect cardiac muscles [21], MLN in skeletal muscles, endoregulin (ELN) in endothelial and epithelial tissues [24], the ubiquitous another-regulin (ALN) [24], and dwarf open reading frame (DWORF) in cardiac and skeletal muscles [23].

Herein, we summarize what is known about the functional properties of PLN, SLN, and DWORF, and we present new functional data for MLN, ELN, and ALN. Comparison of the structural and functional properties of these regulins reveals that they are distinct regulatory subunits of SERCA. PLN and DWORF are present together in cardiac muscles and they have similar structural features [25]. However, DWORF is a smaller peptide with a critical proline residue in the transmembrane domain (Figure 1). These structural differences impart distinct functional properties—PLN is an inhibitor of SERCA and DWORF is the first known peptide activator of SERCA. SLN and MLN are present together in skeletal muscles and their main structural feature is a transmembrane domain. Both SLN and MLN are inhibitors of SERCA, though the mechanisms of inhibition and the functional consequences are distinct. Finally, we present new functional data for ELN and a structural model and new functional data for ALN. The ubiquitous ALN is the largest of the regulin peptides. Considering the functional characteristics of SERCA regulation by each of the regulin peptides, ALN closely resembles the SERCA inhibitory properties of SLN. MLN and ELN resemble one another and possess distinct functional characteristics in the regulation of SERCA. An underlying assumption is that all regulin peptides interact with SERCA in the same “inhibitory groove” [22,23,24]. The different functional properties of the regulins can then be rationalized in terms of their distinct structures and modes of interaction with SERCA, including the unique activation behavior of the DWORF peptide [25].

## 2. Phospholamban (PLN)—The Founding Member of the Regulin Family

PLN is a 52 amino acid transmembrane peptide (Figure 1) that is primarily expressed in the ventricles of the heart, and in lower amounts in atrial muscles, slow-twitch skeletal muscles, and smooth muscles. PLN has been directly linked to dilated cardiomyopathy (DCM) through the identification of hereditary pathological variants. The first pathological variant reported in 2003 was an Arg^9^-Cys missense mutation identified in a large family with a history of DCM and an average age of DCM-related death of 25 years [26]. Around the same time, a Leu^39^ truncation variant was identified, where heterozygous individuals develop hypertrophy and homozygous individuals develop severe DCM and heart failure [27]. Leu^39^ truncation is unusual among the known PLN variants in that it equates to a null phenotype and both heterozygous and homozygous carriers have been found. A third variant was identified in 2006, an Arg^14^-deletion mutation associated with both mild and severe DCM [28,29]. The Arg^14^-del mutation in PLN is the most common mutation identified in DCM patients in the Netherlands [30]. Since the identification of these first genetic variants in PLN, more than 30 genetic variants have since been identified through genetic testing and genome sequencing projects and the role of PLN in normal and diseased myocardium has solidified. The vast majority of the newly identified PLN variants remain uncharacterized with regards to their disease association, and this is a knowledge gap that needs to be filled [31].

**Figure 2:**
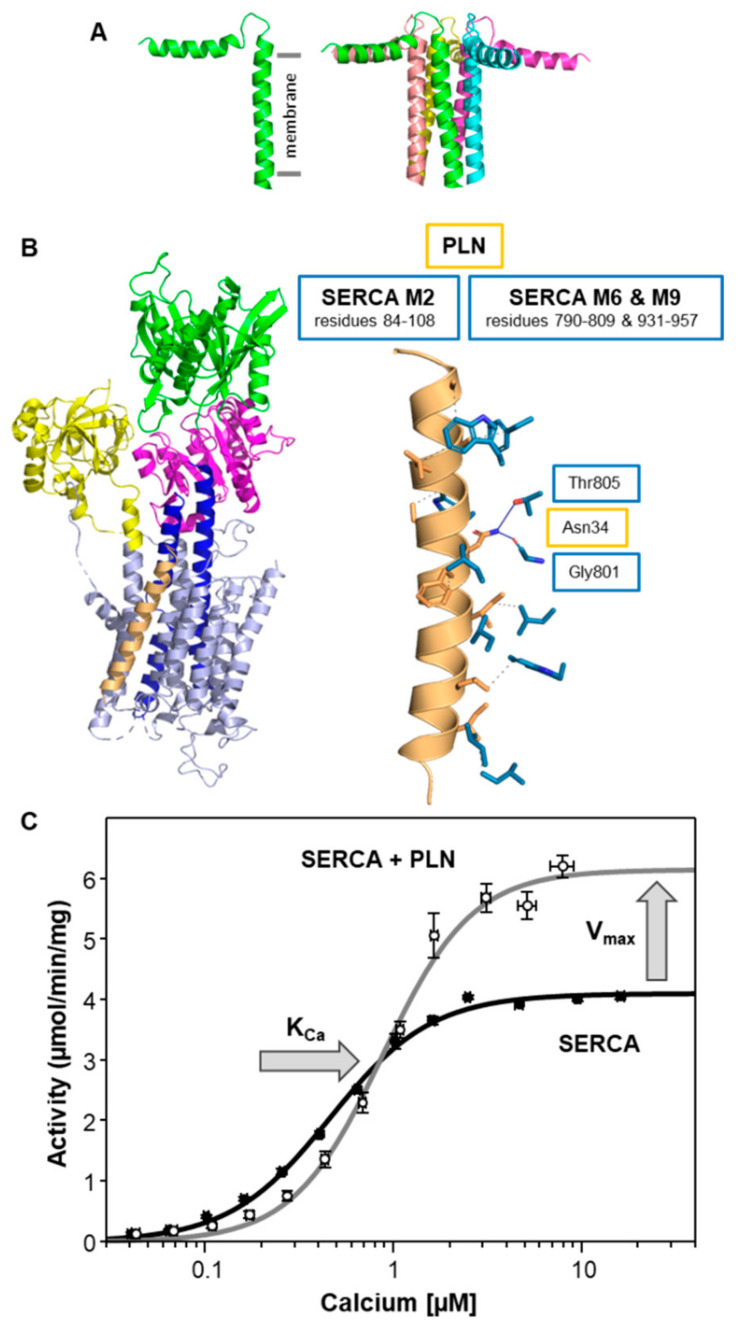
SERCA and phospholamban (PLN). (**A**) Molecular model of the PLN monomer and pentamer (PDB code 2KYV). The transmembrane domain is indicated. (**B**) Molecular model of the SERCA-PLN complex (PDB code 4KYT). PLN is colored tan (chain B; chain C is not shown). SERCA is colored light blue with the actuator domain in yellow, the phosphorylation domain in magenta, the nucleotide-binding domain in green, and transmembrane segments M4 and M5 in blue. Also shown are the side-chain interactions between SERCA (blue) and PLN (tan). The relative locations of SERCA transmembrane segments M2, M6, and M9 are indicated. An essential interaction involving Asn^34^ of PLN and Thr^805^ and Gly^801^ of SERCA is shown. For the full list of interactions, refer to Table S1. (**C**) Calcium-dependent ATPase activity of SERCA reconstituted into membrane vesicles in the absence (black) and presence of PLN (gray). The changes in the apparent calcium affinity (K_Ca_) and maximal activity (V_max_) of SERCA are indicated by arrows. Each data point is the mean ± standard error (*n* ≥ 3).

Over the past decades, extensive studies have examined the structural and functional importance of PLN in regulating the calcium transporting activity of SERCA. Structurally, PLN is a tail-anchored membrane protein that consists of an N-terminal, cytoplasmic helix (residues ~1–18), a flexible linker region (residues ~19–25), and a transmembrane helix (residues ~25–52; Figure 2A) [32]. Functionally, PLN inhibits SERCA by reducing its apparent affinity for calcium (K_Ca_). The crystal structure of the SERCA-PLN complex revealed the transmembrane domain of PLN bound to a calcium-free E1-like state of SERCA [33], where PLN interacts with an inhibitory groove formed by transmembrane segments M2, M6, and M9 of SERCA (Figure 2B). While PLN prefers to interact with calcium-free conformations of SERCA, PLN is known to bind with differential affinity to both the calcium-bound and calcium-free conformations [34]. Inhibition of SERCA is relieved through β-adrenergic signaling and phosphorylation of PLN at Ser^16^ by protein kinase A [12], though PLN can also be phosphorylated at Thr^17^ by CaMKII [13] or protein kinase B (Akt [14]). PLN is known to form oligomers with a monomer and pentamer being dominant species (Figure 2A) [13,20]. The PLN monomer is thought to be the inhibitory species and the primary form that interacts with the inhibitory groove of SERCA. However, the crystal structure of the SERCA-PLN complex revealed a PLN dimer bound to SERCA. The PLN pentamer has been shown to bind to an accessory site on SERCA that is distinct from the inhibitory groove and formed by transmembrane segment M3 [35,36].

The primary target of PLN is the cardiac muscle isoform SERCA2a. However, the tissue distribution of PLN indicates that it is also present with the skeletal muscle isoform SERCA1a and the ubiquitous isoform SERCA2b. PLN has been shown to inhibit all three SERCA isoforms [37,38]. PLN inhibition manifests as a shift in the apparent calcium affinity of SERCA, which saturates at a one-to-one molar stoichiometry [35,39] (Table 1). At higher molar ratios similar to those found in cardiac SR membranes (~4 PLN to 1 SERCA [40]), PLN also increases the maximal activity (V_max_) of SERCA [35,40,41,42]. The net effect (Figure 2C) is inhibition of SERCA at lower calcium concentrations (~0.1 to 1.0 µM calcium) and activation of SERCA at higher calcium concentrations (~1.0 to 10 µM calcium). On the one hand, inhibition of SERCA is due to the interaction of the transmembrane domain of PLN with the inhibitory groove of SERCA (transmembrane segments M2, M6, and M9 [33]). On the other hand, activation of SERCA is attributed to the interaction of PLN with an accessory site of SERCA (transmembrane segment M3 [35,43,44]). One of the most important residues for inhibition of SERCA is Asn^34^ of PLN, where mutation of this residue to alanine causes a complete loss of PLN inhibitory function [41,42,45]. A critical interaction required for inhibition involves Asn^34^ in the transmembrane domain of PLN and Thr^805^ in transmembrane segment M6 of SERCA [33,46] (Figure 2B and Table S1). While PLN interacts with M2, M6, and M9 of SERCA, the interaction with M6 appears to underlie SERCA inhibition and the effect of PLN on the apparent calcium affinity of SERCA (Table 2). Transmembrane segment M6 contributes to calcium binding and this SERCA-PLN interaction slows the structural transition that accompanies the binding of calcium to SERCA. In contrast, the interaction required for activation of SERCA relies on the cytoplasmic amphipathic helix and the pentameric state of PLN [35]. The interaction of the PLN pentamer with transmembrane segment M3 of SERCA results in the coordinated positioning of the cytoplasmic domains of PLN adjacent to the calcium access funnel of SERCA. The underlying mechanism involves modulation of the membrane bilayer and enhancement of the turnover rate of SERCA.

## 3. Sarcolipin (SLN)—A Proteolipid Becomes a Regulin

SLN was first discovered as a proteolipid of unknown function that co-purified with SERCA in skeletal muscle SR membranes [47]. The proteolipid was later identified and named sarcolipin [48]. SLN is a 31 amino acid transmembrane peptide (Figure 1) that is primarily expressed in fast-twitch skeletal muscles, with lower amounts in slow-twitch skeletal muscles and atrial muscles [49]. It is clear that SLN is a key regulator of atrial contractility such that changes in SLN expression levels may result in atrial fibrillation, arrhythmias, and remodeling [50,51]. However, a direct linkage between SLN and the molecular genetics of atrial fibrillation has not yet been found [52]. SLN has also been linked to muscle-based, non-shivering thermogenesis and energy metabolism [11]. This latter role has refocused attention on SLN and the regulation of SR calcium handling in skeletal muscles.

**Figure 3:**
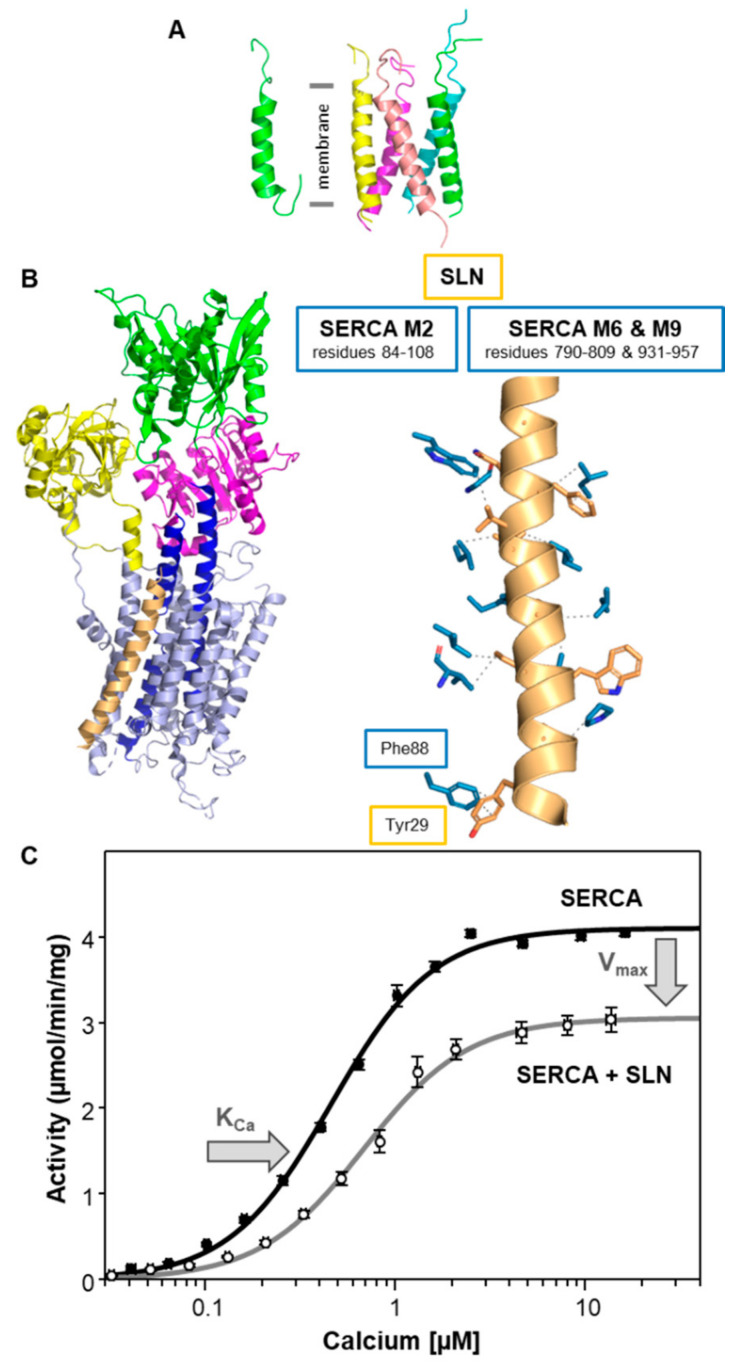
SERCA and sarcolipin (SLN). (**A**) Molecular model of the SLN monomer (PDB code 1JDM) and pentamer [58]. The transmembrane domain is indicated. (**B**) Molecular model of the SERCA-SLN complex (PDB codes 3W5A and 4H1W). SLN is colored tan. SERCA is colored light blue with the actuator domain in yellow, the phosphorylation domain in magenta, the nucleotide-binding domain in green, and transmembrane segments M4 and M5 in blue. Also shown are the side-chain interactions between SERCA (blue) and SLN (tan). The relative locations of SERCA transmembrane segments M2, M6, and M9 are indicated. An interaction involving Tyr^29^ of SLN and Phe^88^ of SERCA is shown. For the full list of interactions, refer to Table S2. (**C**) Calcium-dependent ATPase activity of SERCA reconstituted into membrane vesicles in the absence (black) and presence of SLN (gray). The changes in the apparent calcium affinity (K_Ca_) and maximal activity (V_max_) of SERCA are indicated by arrows. Each data point is the mean ± standard error (*n* ≥ 3).

Early studies of SERCA regulation focused primarily on PLN because of its role in cardiac contractility and the important implications for heart disease. By comparison, fewer studies focused on SLN because of its localization in skeletal muscles and an uncertain relationship with human disease. SLN is functionally similar to PLN in that it inhibits SERCA by reducing its apparent affinity for calcium (K_Ca_), though there are fundamental differences in the structure and function of SLN. Structurally, SLN is a tail-anchored membrane protein that consists of a short, unstructured cytoplasmic domain (residues 1–6), a transmembrane helix (residues 7–26), and a short unstructured domain that resides in the SR lumen (residues 27–31) (Figure 3A). Functionally, SLN inhibition of SERCA relies on a highly conserved luminal tail (C-terminal residues Arg^27^-Ser-Tyr-Gln-Tyr^31^; Figure 1) [53]. This is quite different from PLN inhibition of SERCA, which relies on the transmembrane domain of PLN. Crystal structures of the SERCA-SLN complex revealed SLN bound to the inhibitory groove of SERCA in a calcium-free E1-like conformation [54,55], much like what was observed for the SERCA-PLN complex [33] (Figure 3B). Like PLN, SLN inhibition is associated with binding to the calcium-free conformations of SERCA, but SLN remains associated with the calcium-bound conformations of SERCA [56]. Inhibition of SERCA is relieved by phosphorylation of SLN, though the underlying mechanism is poorly understood. The N-terminus of SLN has a conserved threonine residue (Thr^5^) that appears to be a target for phosphorylation by CaMKII [15] and serine/threonine kinase 16 (STK16 [16]). SLN is known to form a variety of oligomers with a monomer and dimer being the dominant species and the pentamer being less stable compared to PLN [35,57,58] (Figure 3A). SLN has also been shown to bind to the M3 accessory site of SERCA, though the interaction is distinct from PLN and involves both an SLN monomer and pentamer [58].

The primary target of SLN is the skeletal muscle isoform SERCA1a, though the presence of SLN in atrial muscles indicates that it is also present with the cardiac muscle isoform SERCA2a [59]. SLN can also inhibit the SERCA2b and SERCA3a isoforms [24]. SLN inhibition is observed as a shift in the apparent calcium affinity of SERCA at equimolar ratios [53,58] (Table 1). At higher molar ratios (~4 SLN to 1 SERCA), SLN also decreases the maximal activity (V_max_) of SERCA at saturating calcium concentrations (Figure 3C), which is opposite to the behavior observed for PLN (Figure 2C). The expression level of SLN in skeletal muscles is lower than that in this experimental condition (~1.2 SLN per SERCA [60]), though the higher molar ratio may be representative of pathological conditions [61,62,63,64]. Nonetheless, the effect of SLN on the K_Ca_ and V_max_ of SERCA lowers the turnover rate of SERCA at all calcium concentrations (~0.1 to 10 µM calcium; Figure 3C). Inhibition of SERCA relies on the luminal tail of SLN (Arg^27^-Ser-Tyr-Gln-Tyr^31^), where removal of this domain causes a complete loss of SLN inhibitory function [53]. A critical interaction required for inhibition involves Asn^11^ in the transmembrane domain of SLN, which is homologous to Asn^34^ of PLN, and Thr^805^ in transmembrane segment M6 of SERCA [33,46]. In the crystal structures of the SERCA-SLN complex [54,55], the luminal tail of SLN is helical and Tyr^29^ interacts with Phe^88^ on transmembrane segment M2 of SERCA (Figure 3B and Table S2). Like the SERCA-PLN complex, the SERCA-SLN interaction is thought to slow a structural transition that accompanies calcium binding to SERCA. The role of the luminal tail of SLN in this mechanism may involve the correct positioning of SLN’s transmembrane domain in the membrane bilayer and inhibitory groove of SERCA such that the Asn^11^-Thr^805^ interaction can occur. It is notable that both PLN and SLN interact with M2, M6, and M9 of SERCA, and that the interaction with M6 may underlie the inhibitory effect on the apparent calcium affinity of SERCA (Table 2).

With the reported role of SLN in muscle-based, non-shivering thermogenesis [11], an outstanding question in the field is the molecular underpinnings of this process. SLN has been reported to uncouple ATP hydrolysis from calcium transport [65] and thereby increase heat production by SERCA [66]. A possible mechanism for uncoupling has been proposed and may involve the interaction between the N-terminus of SLN and SERCA [67]. We have not explored the uncoupling of SERCA by SLN using our membrane reconstitution system, though our available data indicates that SLN decreases the rate of steady-state ATP hydrolysis by SERCA ([53,58] and Figure 3C), but it does not alter pre-steady state calcium transport by SERCA [68]. At first glance, these data do not seem consistent with uncoupling, though further investigation is warranted.

## 4. Dwarf Open Reading Frame (DWORF)—A Little Regulin with a Big Heart

Dwarf open reading frame (DWORF) is a member of the newly identified regulins, which include myoregulin (MLN), endoregulin (ELN), and another-regulin (ALN). These peptides were found to be encoded by RNAs previously annotated as long noncoding [22,23,24], and they are part of a continuously expanding list of functional micropeptides [17]. Of the newly identified regulins, DWORF has received more research attention due to its expression in cardiac muscles and its potential role in the development, progression, and treatment of heart disease [69,70]. Indeed, gene therapy with the DWORF peptide has been shown to improve outcomes in an animal model of DCM [70]. It has become clear that DWORF is an important new regulator of cardiac contractility. DWORF is a 35 amino acid transmembrane peptide (Figure 1), which is primarily expressed in ventricular and slow-twitch skeletal muscles, and absent in atrial and fast-twitch skeletal muscles [23]. DWORF has been shown to displace PLN from binding to SERCA and thereby enhance SERCA activity [23,69]. In addition to the displacement of PLN, we have shown that DWORF is a direct activator of SERCA even in the absence of PLN [25]. Thus, DWORF activates SERCA and cardiac contractility using a dual mechanism—DWORF relieves SERCA inhibition by displacing PLN and it directly enhances SERCA activity.

**Figure 4:**
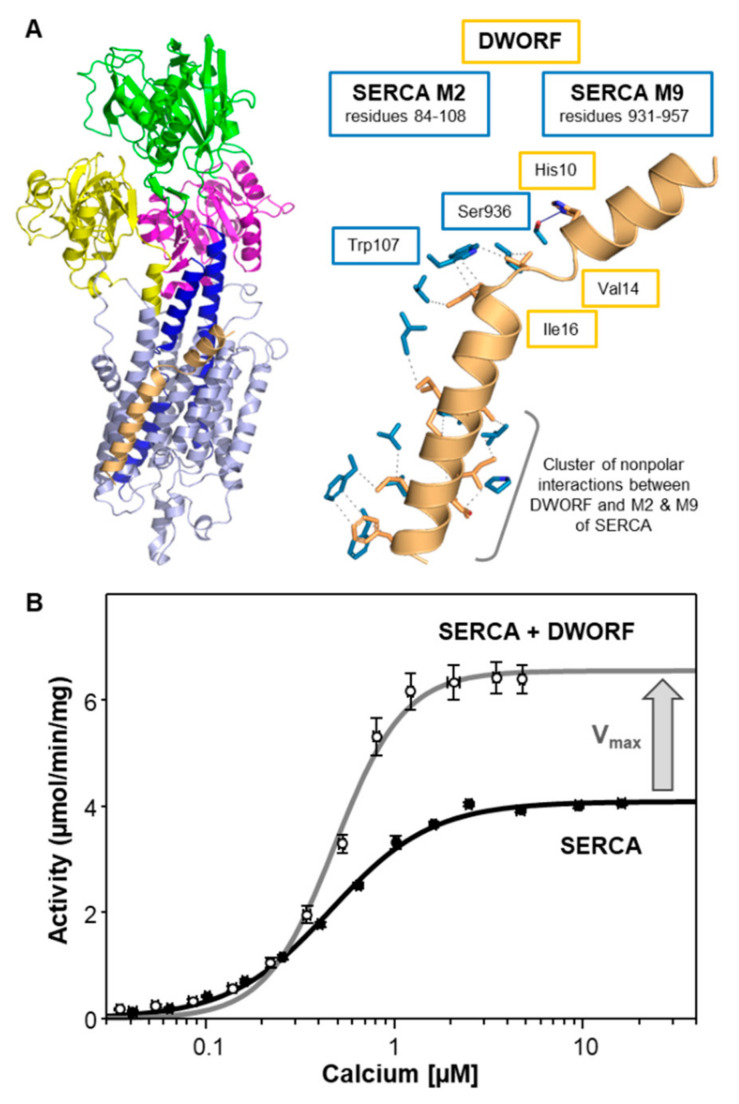
SERCA and dwarf open reading frame (DWORF). (**A**) Molecular model of the SERCA-DWORF complex [25]. DWORF is colored tan. SERCA is colored light blue with the actuator domain in yellow, the phosphorylation domain in magenta, the nucleotide-binding domain in green, and transmembrane segments M4 and M5 in blue. Also shown are the side-chain interactions between SERCA (blue) and DWORF (tan). The relative locations of SERCA transmembrane segments M2 and M9 are indicated. Key interactions between DWORF and SERCA are indicated. For the full list of interactions, refer to Table S3. (**B**) Calcium-dependent ATPase activity of SERCA reconstituted into membrane vesicles in the absence (black) and presence of DWORF (gray). The change in the maximal activity (V_max_) of SERCA is indicated by an arrow. Each data point is the mean ± standard error (*n* ≥ 3).

The structural and functional properties of DWORF are unique among the regulins. Structurally, DWORF consists of a short, cytoplasmic helix (residues 1–13), a flexible linker centered around Pro^15^ (residues 14–16), and a transmembrane helix (residues 17–35) (Figure 4A) [25,71]. Overall, the structural features of DWORF (Figure 4A) are similar to PLN (Figure 2A), though DWORF is a much shorter peptide (35 residues versus 52 residues, respectively). The N-terminal helix of DWORF is amphipathic and reminiscent of the N-terminal helix of PLN. The transmembrane domain of DWORF is disrupted by Pro^15^, which eliminates interactions that are known to be required for SERCA inhibition by PLN (e.g., Asn^34^). This suggests a mechanism for how DWORF displaces PLN from the inhibitory groove of SERCA without also being inhibitory. DWORF can bind to the inhibitory groove of SERCA [23,72], though it lacks structural elements that are known to be required for SERCA inhibition [25]. Functionally, DWORF activation of SERCA is thought to rely on the helix-linker-helix structure of DWORF. A molecular model of the SERCA-DWORF complex places the short transmembrane helix of DWORF in the inhibitory groove of SERCA and the amphipathic helix of DWORF at the membrane surface [25] (Figure 4A). Critical contacts that stabilize the SERCA-DWORF complex include His^10^ on the amphipathic helix of DWORF and Ser^936^ of SERCA (Table S3). Trp^107^ on transmembrane segment M2 of SERCA appears to stabilize the linker region of DWORF (Val^14^-Pro^15^-Ile^16^) and there is a cluster of nonpolar interactions between the transmembrane domain of DWORF and transmembrane segments M2 and M9 of SERCA. Compared to PLN and SLN, DWORF makes more extensive contacts with M2 of SERCA and there appears to be only a single interaction with M6 of SERCA (Table 2). Compared to PLN and SLN, the lack of interaction between DWORF and M6 of SERCA offers an explanation for why DWORF itself is not inhibitory.

The primary target of DWORF is the cardiac muscle isoform SERCA2a, though DWORF can interact with all SERCA isoforms [23]. DWORF activation of SERCA is observed as an increase in the turnover rate and maximal activity (V_max_) of SERCA at equimolar ratios [25] (Figure 4B and Table 1). As previously observed by others, DWORF does not have an effect on the apparent calcium affinity (K_Ca_) of SERCA [23,69]. Since DWORF selectively impacts the V_max_ of SERCA, it is interesting to consider what happens to the V_max_ of SERCA in the presence of PLN and SLN. PLN can increase the V_max_ of SERCA at higher molar ratios (Figure 2C [35]), whereas SLN decreases the V_max_ of SERCA at higher molar ratios (Figure 3C [58]). The higher molar ratio of PLN to SERCA is in the physiological range [40,73,74], though the higher molar ratio of SLN to SERCA appears to be non-physiological [60] or pathophysiological [61,62,63,64]. Nonetheless, the SERCA regulatory subunits found in cardiac muscles, PLN and DWORF, can increase the turnover rate of SERCA, while the skeletal muscle regulatory subunit, SLN, can decrease the turnover rate of SERCA. The increase in SERCA activity appears to be a property of cardiac muscles, and the decrease in SERCA activity appears to be a property of skeletal muscles, as well as the other members of the regulin family (see below).

## 5. Myoregulin (MLN)—The Regulin for Athletes

MLN was discovered through a bioinformatics screen of uncharacterized skeletal muscle RNA transcripts annotated as putative long noncoding [22]. The RNA was found to contain a small open reading frame that encoded a conserved, functional micropeptide. The peptide was named myoregulin (MLN) and it was found to have structural and functional features that are similar to PLN and SLN. MLN is a 46 amino acid transmembrane peptide (Figure 1), and it appears to be the primary SERCA regulatory subunit expressed in adult mouse skeletal muscles [23,24]. Thus, SLN and MLN are present together in skeletal muscles and there is evidence that they have distinct physiological functions. SLN regulation of SERCA appears to play a role in muscle-based thermogenesis [11]. MLN, on the other hand, appears to play a role in skeletal muscle performance. Genetic deletion of MLN in mice enhanced SERCA-dependent calcium handling in skeletal muscles and improved exercise performance—that is, the knockout mice are better athletes [22]. These data suggest that MLN could be a therapeutic target for skeletal muscle diseases where calcium cycling and muscle performance are impaired. By comparison, genetic deletion of SLN in mice reduced muscle-based thermogenesis and energy metabolism [75] and enhanced atrial contractility [50].

**Figure 5:**
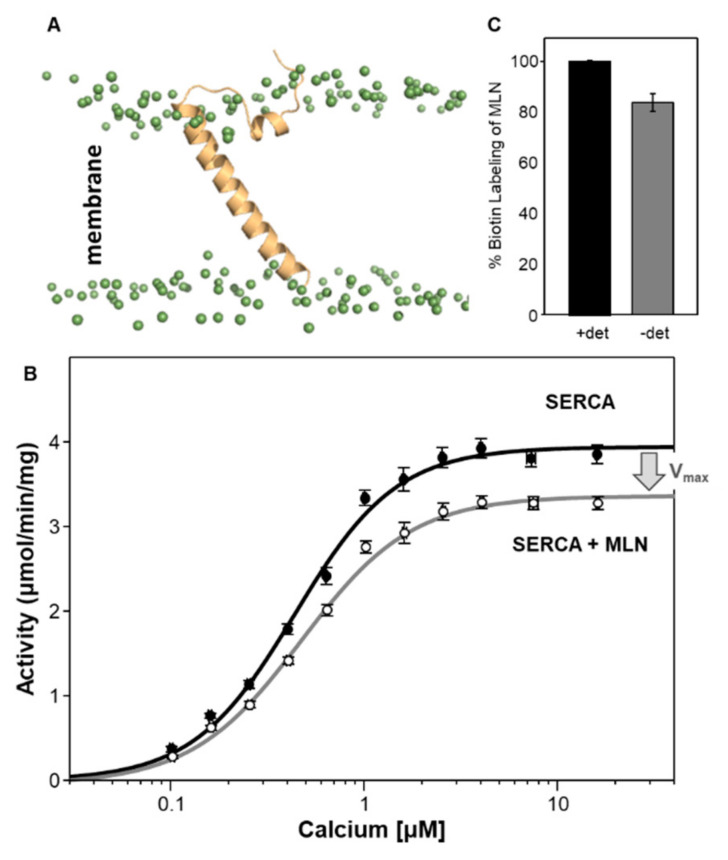
SERCA and myoregulin (MLN). (**A**) Molecular model of MLN in a lipid bilayer. The phospholipid headgroups of the lipids are indicated as green spheres. The N-terminal cytoplasmic domain of MLN (residues 1–16) is largely unstructured and lies along the membrane surface and the transmembrane domain (residues 17–46) is inclined approximately 60° relative to the bilayer surface (~30° relative to the bilayer normal). (**B**) Calcium-dependent ATPase activity of SERCA reconstituted into membrane vesicles in the absence (black) and presence of MLN (gray). The change in the maximal activity (V_max_) of SERCA is indicated by an arrow. Each data point is the mean ± standard error (*n* ≥ 3). (**C**) Biotin labeling and sidedness of MLN in the co-reconstituted proteoliposomes in the absence and presence of detergent. On average, 84 ± 1% of the MLN peptides are correctly oriented with their cytoplasmic domains on the exterior surface of the co-reconstituted proteoliposomes.

Much like DWORF, the structural and functional properties of MLN are distinct. Structurally, MLN consists of an unstructured cytoplasmic domain that lies along the membrane surface (residues ~1–17) and an extended transmembrane helix (residues 18–46) (Figure 5A). The predicted transmembrane domain encompasses residues Ile^21^ to Val^43^ (TMHMM [76]); however, molecular dynamics simulations of MLN in a lipid bilayer indicate a more extended transmembrane helix that is inclined approximately 60° relative to the bilayer surface (~30° relative to the bilayer normal). Functionally, MLN has been reported to alter the apparent calcium affinity of SERCA in homogenates from HEK 293 cells expressing both proteins [22]. Using a membrane reconstitution system, we have found that MLN selectively alters the V_max_ of SERCA with no effect on the K_Ca_ of SERCA (Figure 5B and Table 1). The difference between these two observations may lie in the experimental approach: (i) heterologous expression in HEK 293 cells versus purified proteins and membrane reconstitution, (ii) the measurement of calcium transport versus ATPase activity, and (iii) the orientation of MLN in the reconstituted membrane vesicles. Membrane reconstitution provides a simplified system that allows detailed mechanistic insight into SERCA regulation by MLN, though it lacks the many calcium-handling factors present in a cellular environment that may contribute to calcium homeostasis. We have previously compared calcium transport and ATPase activity of SERCA-PLN proteoliposomes and found them to be comparable [68]. We have also measured the orientation of PLN [41] and MLN (Figure 5C) in the reconstituted proteoliposomes and found them to be properly oriented with their cytoplasmic domains on the exterior surface of the membrane vesicles. Of note, previous studies of the PLN Arg^14^-deletion mutant revealed different conclusions using HEK 293 cells (gain of function [29]) versus membrane reconstitution (partial loss of function [40,77]).

**Figure 6:**
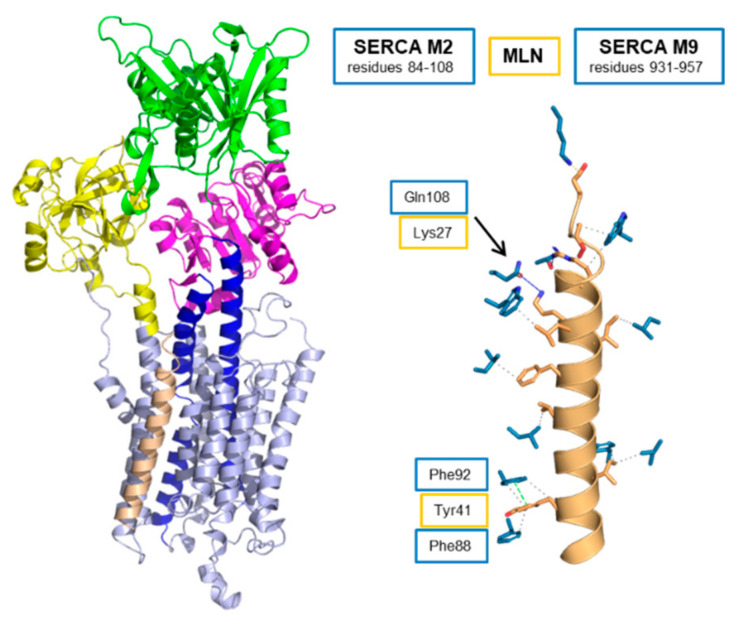
Molecular model of the SERCA-MLN complex. MLN is colored tan. SERCA is colored light blue with the actuator domain in yellow, the phosphorylation domain in magenta, the nucleotide-binding domain in green, and transmembrane segments M4 and M5 in blue. Also shown are Table 2. and M9 are indicated. Interactions involving Lys^27^ of MLN and Gln^108^ of SERCA, and Tyr^41^ of MLN and Phe^88^ and Phe^92^ of SERCA are shown. For the full list of interactions, refer to Table S4.

MLN is thought to interact with the inhibitory groove of SERCA and adopt the same general structure and function as PLN and SLN [22]. We have used molecular modeling and molecular dynamics simulations to construct a model of the SERCA-MLN complex (Figure 6). The sequence alignment used to construct the model placed Lys^27^ of MLN at the position occupied by Asn^34^ of PLN [78], and this sequence alignment differs from the initial report [22]. Asn^34^ is known to be essential for PLN function and to form an important interaction with Thr^805^ of SERCA. We expect a polar residue in MLN to fill this role (e.g., Arg^24^ or Lys^27^). In addition, MLN and PLN are tail-anchored membrane proteins that share structural and functional features. There should be a correlation between their sequence alignment and how they span the membrane bilayer (TMHMM [76] predicts transmembrane residues 21–43 for MLN and 32–51 for PLN). This places Lys^27^ of MLN as the nearest polar residue to the position occupied by Asn^34^ of PLN [78] (Figure 1). In the SERCA-MLN model, MLN primarily contacts transmembrane segments M2 and M9 of SERCA (Figure 6 and Table 2). Key interactions appear to be Lys^27^ of MLN with Gln^108^ of M2, Arg^24^ of MLN with Thr^805^ of M6, and Tyr^41^ of MLN with Phe^88^ and Phe^92^ of M2 (Table S4). Arg^24^ is in contact with Thr^805^ of SERCA, though it appears to be the only contact between MLN and M6 of SERCA. Like DWORF, MLN primarily interacts with transmembrane segments M2 and M9 of SERCA and it makes a single contact with M6 (Table 2). This offers an explanation for why MLN does not alter the apparent calcium affinity of SERCA since inhibition of SERCA by PLN and SLN involves substantial interactions with transmembrane segment M6 of SERCA.

## 6. Endoregulin (ELN) and Another-Regulin (ALN)—Non-Muscle Cells Finally Get Their Turn

Even with the discovery of the new regulins MLN [22] and DWORF [23], peptide regulatory subunits of SERCA were thought to be muscle-specific adaptations of calcium homeostasis—PLN and DWORF in cardiac muscles and SLN and MLN in skeletal muscles. However, SERCA performs essential functions in all cells, and the requirement for regulation is not restricted to muscle cells. There is precedent for widespread regulation of SERCA in the form of the ubiquitous SERCA2b isoform, which encodes its own regulatory domain. The same group that identified MLN and DWORF as functional regulators of SERCA also carried out a bioinformatics screen of the mouse genome for uncharacterized peptides with sequence homology to the other regulins [24]. Two genes were identified and found to encode transmembrane peptides that regulate SERCA in non-muscle tissues. One peptide localized with SERCA3a in endothelial and epithelial tissues (endoregulin; ELN), while the other peptide localized with SERCA2b in a ubiquitous expression pattern (another-regulin; ALN). There are no current physiological or disease-associated roles for ELN or ALN, though the identification of SERCA regulators in non-muscle tissues is an exciting development in our understanding of the regulation of calcium homeostasis in all cells. 

**Figure 7:**
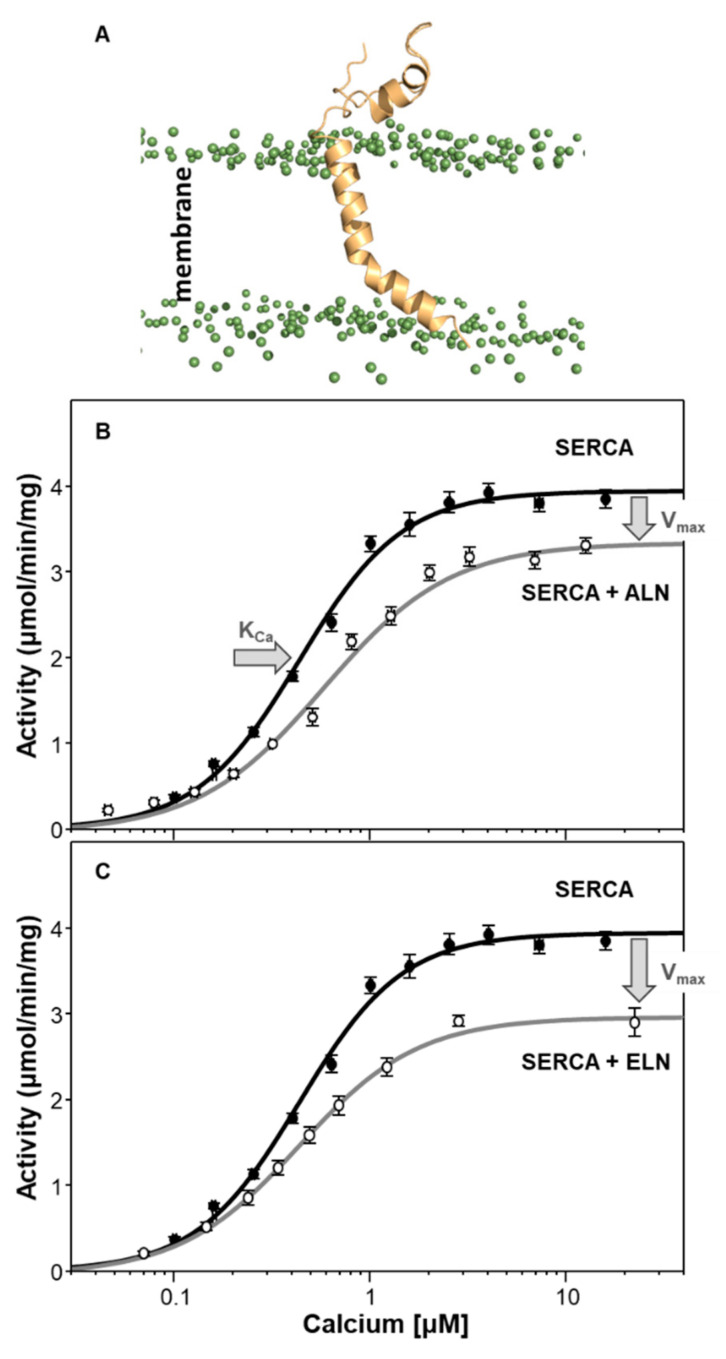
SERCA, another-regulin (ALN) and endoregulin (ELN). (**A**) Molecular model after molecular dynamics simulation of ALN in a lipid bilayer. The headgroups of the phospholipids are indicated as green spheres. The cytoplasmic domain of ALN (residues 1–38) is largely unstructured and lies along the membrane surface. The transmembrane domain of ALN is kinked and slightly longer than predicted (residues 39–65). (**B**) Calcium-dependent ATPase activity of SERCA reconstituted into membrane vesicles in the absence (black) and presence of ALN (gray). The change in the apparent calcium affinity (K_Ca_) and maximal activity (V_max_) of SERCA is indicated by arrows. (**C**) Calcium-dependent ATPase activity of SERCA reconstituted into membrane vesicles in the absence (black) and presence of ELN (gray). The change in the maximal activity (V_max_) of SERCA is indicated by an arrow. Each data point is the mean ± standard error (*n* ≥ 3).

Like their muscle-specific counterparts, ELN and ALN localize to the ER membrane and their helical transmembrane domains are thought to interact with the inhibitory groove of SERCA. The primary target of ELN is the SERCA3 isoform and the primary target of ALN is SERCA2b. ALN has also been detected in skeletal and cardiac muscles, suggesting that it also interacts with the SERCA1a and SERC2a isoforms. Herein, we have characterized the structure and function of ALN, and we present an initial evaluation of the function of ELN. The following discussion will mainly focus on ALN. Structurally, ALN is the largest regulin (66 amino acid peptide) with a long inherently unstructured cytoplasmic domain (residues ~1–42) and a transmembrane domain (TMHMM [76] predicts residues 43–65) (Figure 1). ELN is a 62 amino acid peptide with a cytoplasmic domain (residues 1–25), a predicted transmembrane domain (TMHMM [76] predicts residues 26–48), and a luminal tail (residues 49–62) (Figure 1). Molecular dynamics simulations of ALN in a lipid bilayer revealed an unstructured cytoplasmic domain that lies along the membrane surface and a kinked transmembrane domain that is slightly longer than predicted (residues 39–65) (Figure 7A). We have not yet performed molecular dynamics simulations of ELN in a lipid bilayer. Functional analysis using a membrane reconstitution system revealed that ALN affects both the apparent calcium affinity (K_Ca_) and maximal activity (V_max_) of SERCA (Figure 7B). Of all the regulins, ALN functionally resembled SLN in that it lowered both the K_Ca_ and V_max_ of SERCA (Table 1). We also characterized the regulation of SERCA by ELN using a membrane reconstitution system (Figure 7C and Table 1). ELN was functionally similar to MLN in that it did not impact the K_Ca_ and it lowered the V_max_ of SERCA. It should be noted that our reconstitution system used SERCA1a, while the primary targets of ALN and ELN are SERCA2b and SERCA3a, respectively. However, it has been shown that ALN and ELN regulate SERCA2b and SERCA3a in a similar manner [24], and our data with SERCA1a are consistent with these observations.

**Figure 8:**
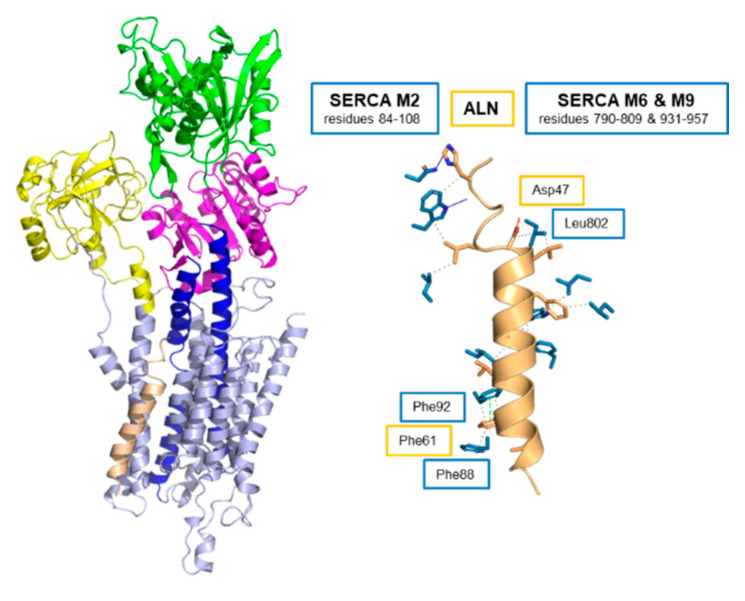
Molecular model of the SERCA-ALN complex. ALN is colored tan. SERCA is colored light blue with the actuator domain in yellow, the phosphorylation domain in magenta, the nucleotide-binding domain in green, and transmembrane segments M4 and M5 in blue. Also shown are the side-chain interactions between SERCA (blue) and ALN (tan). The relative locations of SERCA transmembrane segments M2, M6, and M9 are indicated. Interactions involving Asp^47^ of ALN and Leu^802^ of SERCA, and Phe^61^ of ALN and Phe^88^ and Phe^92^ of SERCA are shown. For the full list of interactions, refer to Table S5.

Like the other regulins, ALN is thought to interact with the inhibitory groove of SERCA similar to PLN and SLN [22]. We used molecular modeling and molecular dynamics simulations to construct a model of the SERCA-ALN complex (Figure 8). The sequence alignment used to construct the model placed Asp^47^ of ALN at the position occupied by Asn^34^ of PLN [22,78] and the transmembrane domain of ALN was modeled as an α-helix. As described above, we expect a polar residue in ALN to fill the role of Asn^34^ in PLN, and we expect maximum overlap of the residues that comprise the transmembrane domain of ALN with the other regulins (Figure 1). Given that the regulins are tail-anchored membrane proteins, there should be a correlation between their sequence alignment and how they span the membrane bilayer (TMHMM [76] predicts transmembrane residues 43–66 for ALN). In the SERCA-ALN model, there is significant unwinding of the transmembrane helix of ALN such that residues 47–63 remain helical. ALN primarily contacts transmembrane segment M2 of SERCA with fewer contacts between ALN and M6 and M9 (Figure 8 and Table 2). Key interactions appear to be Asp^47^ of ALN, which caps the helical region of the transmembrane domain, and Leu^802^ of M6, as well as Phe^61^ of ALN with Phe^88^ and Phe^92^ of M2 (Table S5). His^42^ is in contact with Asn^111^ and Trp^107^ on M2 of SERCA. Like PLN and SLN, ALN makes contacts with the transmembrane segment M6 of SERCA (Table 2), which offers an explanation for why ALN alters the apparent calcium affinity of SERCA in a manner similar to PLN and SLN.

## 7. There Is Nothing Regular about the Regulins

The regulins are a collection of six small transmembrane peptides that act as tissue-specific regulatory subunits of the SERCA calcium pumps. While the existence of “proteolipids” such as PLN and SLN has been known since the 1970′s [20,47], the remaining members of the regulin family were only recently discovered (MLN in 2015 [22]) and (DWORF, ELN, and ALN in 2016 [23,24]). Each regulin has distinct functional properties, which presumably contribute to the unique calcium-handling requirements of different cell types and tissues. The best-studied members of the regulin family, PLN and SLN, are the classic SERCA regulators found in cardiac and skeletal muscles. They “inhibit” SERCA by altering the apparent calcium affinity of the pump (Table 1). At higher molar ratios of PLN-to-SERCA and SLN-to-SERCA, they also alter the turnover rate or maximal activity (V_max_) of SERCA (Figure 2C and Figure 3C). Under these conditions, PLN increases the V_max_ of SERCA and SLN decreases the V_max_ of SERCA.

**Figure 9:**
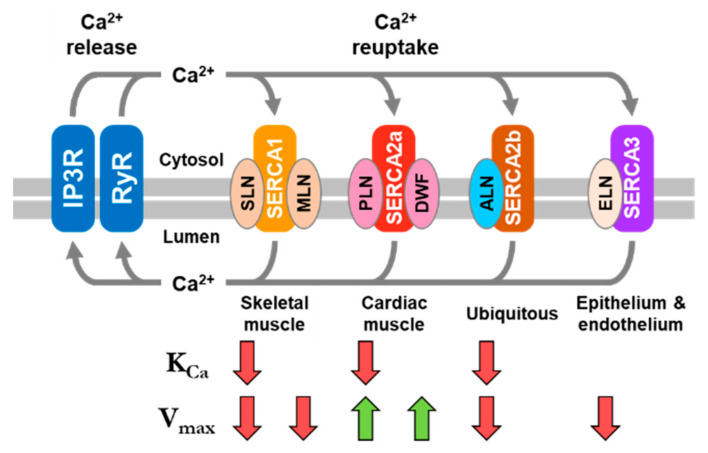
Schematic representation of the expression patterns of the regulins and SERCA isoforms in different muscle and non-muscle tissues in vertebrates (adapted from [24]). The effects of the different regulins on the apparent calcium affinity (K_Ca_) and maximal activity (V_max_) of SERCA are indicated by arrows. Red arrows indicate a decrease in the apparent calcium affinity (K_Ca_) or maximal activity (V_max_), green arrows indicate an increase in the maximal activity (V_max_), no arrows indicate no effect on the apparent calcium affinity (K_Ca_) of SERCA.

Considering the functional properties of PLN and SLN, how do the other regulins compare (Figure 9)? DWORF stands out as the most unique regulin, given that it is the only known peptide activator of SERCA [25]. DWORF and PLN are present together in cardiac muscles, so it is unlikely that they represent redundant functions. Indeed, a dual mechanism has been proposed for DWORF involving the displacement of PLN from the inhibitory groove of SERCA [23,69] and the direct enhancement of SERCA activity (Figure 4B and [25]). Thus, PLN and DWORF encode opposing inhibition and activation functions. It is interesting to note that both DWORF and PLN, the two peptide regulators found in cardiac muscles, are capable of enhancing the turnover rate of SERCA [25,35]. However, excess PLN is required for SERCA activation, while DWORF is capable of SERCA activation under equimolar conditions.

MLN remains a bit of a conundrum in that it was the first of the new regulins to be discovered, yet it was found in skeletal muscles where SERCA is already regulated by SLN. However, MLN and SLN are differentially expressed in slow versus fast-twitch muscle types, and MLN appears to be more abundant with SERCA1a in adult skeletal muscle. Nonetheless, MLN and SLN are found together in some muscle types, and it is unlikely that they represent redundant SERCA regulatory functions. MLN has been reported to alter the apparent calcium affinity (K_Ca_) of SERCA in a manner similar to PLN and SLN [22]. We find that MLN is a selective regulator of the V_max_ of SERCA (Figure 5B). Thus, MLN and SLN may represent separate regulators of the calcium affinity (K_Ca_) and turnover rate (V_max_) of SERCA. The prevalence of MLN in adult skeletal muscles and its ability to reduce the turnover rate of SERCA offers a plausible explanation for the role of MLN in skeletal muscle performance—that is, genetic deletion of MLN in mice enhances SERCA-dependent calcium handling in skeletal muscles and improves exercise performance [22].

The discovery of ALN and ELN in non-muscle tissues laid to rest the concept that SERCA regulation was a muscle-specific adaptation of cellular calcium homeostasis. ALN was found to have a ubiquitous expression pattern, while ELN was found to be expressed in endothelial and epithelial tissues. While the physiological roles of ELN and ALN remain unknown, these peptides offer the first glimpses into SERCA regulation in non-muscle tissues. ELN and ALN have been reported to alter the apparent calcium affinity (K_Ca_) of SERCA in a manner similar to PLN and SLN [24]. We find that ALN alters both the apparent calcium affinity (K_Ca_) and turnover rate (V_max_) of SERCA (Figure 7B), whereas ELN selectively alters the turnover rate (V_max_) of SERCA (Figure 7C). It will be interesting to see how these peptides fit into the SERCA-dependent calcium handling requirements of these tissue types.

Molecular models of the SERCA-regulin complexes allowed us to formulate hypotheses on how the different regulins alter particular transport parameters of SERCA (K_Ca_ versus V_max_). The functional properties of the regulins, as described above, are summarized in Table 1 and Figure 9. The most common behavior of the regulins was a slight depression of the V_max_ of SERCA, which was observed for SLN, MLN, ALN, and ELN. We speculate that this is the default outcome for peptides binding to the inhibitory groove of SERCA formed by transmembrane segments M2, M6, and M9. Peptide binding to this groove impedes the movement of SERCA’s transmembrane domain that is required for calcium binding and progression through the calcium transport cycle. As a result, the peptides slow the turnover rate of SERCA, which manifests as a decrease in V_max_. Another common behavior of the regulins was a decrease in the apparent calcium affinity of SERCA, which was observed for PLN, SLN, and ALN. In the molecular models, the peptides that alter the K_Ca_ of SERCA have more extensive contacts with transmembrane segment M6 of SERCA (Table 2), one of the transmembrane segments involved in calcium binding to SERCA [79]. As a result of the interaction with M6, the peptides impede the conformational change that accompanies the binding of calcium to SERCA. Finally, a less common behavior of the regulins was an increase in the maximal activity of SERCA, which was observed for PLN and DWORF. In molecular models for the SERCA-DWORF (Figure 3B) and SERCA-PLN [35] complexes, the amphipathic helices of the peptides modulate the lipid bilayer and provide a more fluid environment capable of enhancing the turnover rate of SERCA [25,35]. To summarize, we conclude that the structural properties of the regulins and their differential interactions with SERCA provide a spectrum of functional outcomes that cater to the calcium handling requirements of specific cell and tissue types.

## 8. Materials and Methods

All reagents were of the highest purity available: octaethylene glycol monododecyl ether (C_12_E_8_; Nikko Chemicals Co. Ltd., Tokyo, Japan); egg yolk phosphatidylcholine (EYPC), phosphatidylethanolamine (EYPE), and phosphatidic acid (EYPA) (Avanti Polar Lipids, Alabaster, AL, USA); and all reagents used in the coupled enzyme assay including NADH, ATP, PEP, lactate dehydrogenase, and pyruvate kinase (Sigma-Aldrich, Oakville, ON Canada).

### 8.1. Co-Reconstitution of Regulin Peptides with SERCA

With the exception of MLN, recombinant human peptides were expressed as a maltose-binding protein (MBP) fusion with a TEV cleavage site for removal of MBP as previously described [80]. The peptides were purified by a combination of organic extraction (chloroform-isopropanol-water) and reverse-phase HPLC. MLN was ordered as a synthetic peptide (Peptide 2.0 Inc., Chantilly, VA, USA). Purified peptides were stored as lyophilized thin films (100 µg aliquots). SERCA1a was purified from rabbit skeletal muscle SR and this isoform was used for all functional measurements. For co-reconstitution, a lyophilized peptide was suspended in a 100 µL mixture of trifluoroethanol-water (5:1) and mixed with lipids (360 µg EYPC and 40 µg EYPA) from stock chloroform solutions. The peptide-lipid mixture was dried to a thin film under nitrogen gas and placed under a vacuum overnight. The peptide-lipid mixture was rehydrated in buffer (20 mM imidazole pH 7.0; 100 mM NaCl; 0.02% NaN_3_; 50 °C for 15 min). After cooling to room temperature, the peptide-lipid mixture was solubilized in detergent (0.2% C_12_E_8_). Detergent-solubilized SERCA1a was added (300 µg in a total volume of 300 µL) and the reconstitution was stirred gently at room temperature. Detergent was slowly removed by the addition of SM-2 Bio-Beads (Bio-Rad, Hercules, CA, USA) over a 4-h time course (final weight ratio of 25 Bio-Beads to 1 detergent). Following detergent removal, the reconstitution was centrifuged over a sucrose step gradient (20% and 50% layers) for 1 h at 100,000× *g*. The reconstituted proteoliposomes at the gradient interface were removed, flash-frozen in liquid nitrogen, and stored at −80 °C. The final molar ratios ranged from 2–5 peptides per SERCA.

### 8.2. Activity Assays

The reconstituted proteoliposomes contain a high density of SERCA and regulin peptides (low lipid-to-protein ratio) designed to mimic native ER/SR membranes. The resultant proteoliposomes allow the measurement of pre-steady state calcium transport [68] and steady-state ATPase activity (e.g., [40]). In our previous studies of SERCA-PLN, we have found the kinetic parameters from pre-steady state calcium transport and steady-state ATPase activity to be comparable [68]. In this study, the calcium-dependent ATPase activity of the co-reconstituted proteoliposomes was measured by a coupled-enzyme assay as previously described [35,41,42,58]. Data points were collected at 340 nm wavelength, with a well volume of 155 μL containing 10–20 nM SERCA at 30 °C (data points collected every 28–39 s for 20 min). The reactions were initiated by the addition of proteoliposomes to the assay solution. The V_max_ (maximal activity) and K_Ca_ (apparent calcium affinity) were determined based on non-linear least-squares fitting of the activity data to the Hill equation (Sigma Plot software, SPSS Inc., Chicago, IL, USA). Errors were calculated as the standard error of the mean for a minimum of four independent reconstitutions.

### 8.3. Orientation Assay

The orientation of MLN in the co-reconstituted proteoliposomes was determined using a biotin surface labeling assay as previously described [41]. This assay took advantage of the lysine residues in the cytoplasmic domain of MLN and their relative accessibility to labeling in the absence and presence of detergent. SERCA-MLN proteoliposomes (~5 μg of protein) were mixed in labeling buffer (20 mM borate-KOH, pH 9.0) with 5 mM EZ-Link Sulfo-NHS-LC-Biotin (Thermo Fisher Scientific, Waltham, MA, USA) in the absence and presence of 0.5% detergent (*n*-octyl glucopyranoside). The mixture was incubated at 4 °C for 2 h followed by quenching with an equal volume of SDS-PAGE sample buffer. After SDS-PAGE and electroblotting to Immuno-Blot PVDF membranes (Bio-Rad Laboratories, Inc., Hercules, CA, USA), the amount of biotin labeling was quantified using IRDye 800CW streptavidin conjugate and an Odyssey Infrared Imaging System (LI-COR Biosciences, Lincoln, NE, USA).

### 8.4. Molecular Modeling of SERCA-Regulin Complexes

The molecular models of the SERCA-PLN and SERCA-SLN complexes used in this study were the published crystal structures [33,54,55]. The SERCA isoform in both crystals structures is the skeletal muscle SERCA1a isoform. The molecular model of the SERCA-DWORF complex has been previously published [25]. For the regulins MLN and ALN, the protein structure homology-modeling program MODELLER [81] was used to generate molecular models of the peptides as continuous α-helices. Only the transmembrane domains of the regulins predicted by TMHMM [76] were modeled. The SERCA-regulin complexes were modeled based on the SERCA-PLN structure (PDB: 4KYT [33]), the helical models of the transmembrane domains, and the sequence alignments in Figure 1 [78]. Note that the SERCA1a isoform was used in the determination of the SERCA-PLN structure.

### 8.5. Molecular Dynamics Simulations SERCA-Regulin Complexes

Molecular dynamics simulations were carried out as previously described [25,35,58]. Full-length human MLN and ALN were modeled as continuous α-helices. The SERCA-MLN and SERCA-ALN models were constructed as described above. The peptide and complex models were inserted in a 1-palmitoyl-2-oleoyl-sn-glycero-3-phosphocholine (POPC) lipid bilayer containing a total of 180 or 370 lipid molecules, respectively. The systems were solvated, chloride and sodium ions were added to neutralize the total charge of the system (150 mM) and molecular dynamics (MD) simulations were carried out as described [25,35,58]. The MLN and ALN models were subjected to 1 µs MD simulation and the fully equilibrated structures at the end of the simulation are shown (Figure 5 and Figure 7). The SERCA-MLN and SERCA-ALN complexes were subjected to 25 ns MD simulations. The short simulation time was used to preserve comparison to the SERCA-PLN complex while allowing steric clashes and unstable helical regions to equilibrate. To evaluate the interactions between the regulins and SERCA, the Protein Ligand Interaction Profiler was used [82]. PLIP is a web tool for the identification of non-covalent interactions between biological macromolecules (SERCA) and their ligands (regulins). Figure 2B, Figure 3B, Figure 4A, Figure 6 and Figure 8 were generated with Pymol v1.7 [83] and the output files from PLIP.

## Figures and Tables

**Table 1:** Kinetic parameters for SERCA in the absence and presence of regulin peptides.

Regulin	K_Ca_ (µM Calcium)	V_max_ (µmol/min/mg)
SERCA alone	0.44 ± 0.02	4.0 ± 0.1
PLN	0.89 ± 0.03 *^a^*	6.1 ± 0.2 *^a^*
SLN	0.74 ± 0.03 *^a^*	3.1 ± 0.1 *^a^*
DWORF	0.48 ± 0.03	6.9 ± 0.1 *^a^*
MLN	0.47 ± 0.02	3.4 ± 0.1 *^a^*
ALN	0.58 ± 0.05 *^b^*	3.4 ± 0.1 *^a^*
ELN	0.44 ± 0.02	3.1 ± 0.1 *^a^*

*^a^ p* < 0.01 compared with SERCA in the absence of regulin peptides. *^b^ p* < 0.05 compared with SERCA in the absence of regulin peptides.

**Table 2:** Number of molecular contacts between the regulins and SERCA transmembrane segments M2, M6, and M9.

Regulin	Number of SERCA Contacts		
	M2	M6	M9
PLN	1	5	7
SLN	4	6	6
DWORF	9	1	7
MLN	6	1	5
ALN	10	3	3

## Data Availability

The data presented in this study are available on request from the corresponding author.

## Supplementary Materials

The following are available online at https://www.mdpi.com/article/10.3390/ijms22168891/s1.
