# Supplementary material for: Nothing Regular about the Regulins: Distinct Functional Properties of SERCA Transmembrane Peptide Regulatory Subunits
Source: Int J Mol Sci. 2021 Aug 18;22(16):8891. doi: 10.3390/ijms22168891 (PMC8396278; doi:10.3390/ijms22168891)
Supplement: Supplementary file 1 [file ijms-22-08891-s001.zip › ijms-1331047-supplementary.pdf]

International Journal of
Molecular Sciences

MDPI

Table S1: Molecular contacts between phospholamban (PLN) and SERCA.

**PLN residues**	**SERCA residues**		
	**M2**	**M6**	**M9**
Ala24			Trp932
Ala27		Phe809	Trp932
Leu28			Trp932
Ile33	Trp107		
Asn34		Gly801, Thr805	
Phe35			Leu939
Ile38		Leu797	Leu943
Leu42		Trp794	
Ile45			Leu953, Ile956
**13 contacts:** 1 with M2, 5 with M6, and 7 with M9			

*Based on the reported crystal structure of the SERCA-PLN complex (PDB code 4KYT).

Table S2: Molecular contacts between sarcolipin (SLN) and SERCA.

**SLN residues**	**SERCA residues**		
	**M2**	**M6**	**M9**
Leu8		Ala806	
Asn11		Gly801, Thr805	
Phe12		Leu802	Leu939
Val14	Trp107		
Val15		Leu797	Leu943
Val19		Trp794	Leu946, Leu953
Ile22	Val93, Leu96		
Trp23			Leu953
Val26			Pro952
Tyr29	Phe88		
**15 contacts:** 4 with M2, 5 with M6, and 6 with M9			

*Based on the reported crystal structures of the SERCA-SLN complex (PDB codes 3W5A and 4H1W).

Table S3: Molecular contacts between dwarf open reading frame (DWORF) and SERCA.

**DWORF residues**	**SERCA residues**		
	**M2**	**M6**	**M9**
His11			Ser936
Val14	Trp107	Leu802	
Ile16	Val104, Trp107		
Ile23	Ile103		
Val24			Leu953
Ile27			Ile956, Phe957
Ile28			Leu953
Ile30	Val89, Phe92, Val93		
Tyr31			Pro952, Ile956
Phe34	Phe88, Phe92		
**17 contacts:** 9 with M2, 1 with M6, and 7 with M9			

*Based on previously published molecular model of the SERCA-DWORF complex (Fisher et al., *eLife*, 2021).

Table S4: Molecular contacts between myoregulin (MLN) and SERCA.

MLN residues	SERCA residues		
	**M2**	**M6**	**M9**
Glu20			Trp932
Arg24		Thr805	Trp932
Leu26	Trp107		
Lys27	Gln108		
Ile28			Leu939
Phe30	Ile103		
Val34	Leu96		
Ile39			Leu953, Phe957
Tyr41	Phe88, Phe92		
**12 contacts:** 6 with M2, 1 with M6, and 5 with M9			

*Based on the molecular model shown in Figure 6.

Table S5: Molecular contacts between another-regulin (ALN) and SERCA.

ALN residues	SERCA residues		
	**M2**	**M6**	**M9**
His42	Trp107, Asn111		
Leu46	Ile103, Trp107		
Asp47		Leu802	
Leu48		Leu802	
Phe51			Leu943, Leu946
Phe54	Leu96	Trp794	Phe957
Val57	Leu96		
Phe61	Val89, Phe88, Phe92		
Leu65	Val89		
**18 contacts:** 10 with M2, 3 with M6, and 3 with M9			

*Based on the molecular model shown in Figure 8.
